# Primary pulmonary small cell carcinoma in a 13-year-old boy with prolonged second-hand tobacco smoke exposure: A rare pediatric lung malignancy with a review of reported cases and registry data

**DOI:** 10.1016/j.rmcr.2026.102533

**Published:** 2026-10-19

**Authors:** Seyedeh Zalfa Modarresi, Mahla Towhidifar, Somayeh Talaeipour, Maryamsadat Yazdanparast, Fariba Binesh, FatemehalSadat AghaeiMeybodi

**Affiliations:** a Children Growth Disorder Research Center, Comprehensive Research Institute for Maternal and Child Health, Shahid Sadoughi University of Medical Sciences, Yazd, Iran; b Mother and Newborn Health Research Center, Comprehensive Research Institute for Maternal and Child Health, Shahid Sadoughi University of Medical Sciences, Yazd, Iran; c Student Research Committee, Shahid Sadoughi University of Medical Sciences, Yazd, Iran; d Hematology and Oncology Research Center, Non-Communicable Diseases Research Institute, Shahid Sadoughi University of Medical Sciences, Yazd, Iran; e Department of Pathology, Shahid Sadoghi University of Medical Sciences, Yazd, Iran; f Department of Internal Medicine, Shahid Sadoughi University of Medical Sciences, Yazd, Iran

**Keywords:** Primary pulmonary small cell carcinoma, Environmental tobacco smoke, Adolescent, Case report

## Abstract

**Background:**

Primary pulmonary small cell carcinoma (PPSCC) is an exceptionally rare, highly aggressive neuroendocrine carcinoma in the pediatric population, with only a handful of cases reported worldwide. Its rarity, nonspecific clinical presentation, and limited published experience often delay diagnosis and leave important questions regarding its clinicopathological characteristics and potential risk factors unanswered.

**Case presentation:**

A 13-year-old boy presented with a four-month history of persistent cough, progressive dyspnea, recurrent lower respiratory tract infections, and significant unintentional weight loss despite treatment for presumed asthma and pneumonia. Shortly before admission, he developed right parasternal chest pain. He had no history of active smoking; however, he had a prolonged history of exposure to environmental tobacco smoke at home (heavy paternal smoking). Contrast-enhanced chest computed tomography revealed a large right hilar mass with mediastinal extension, bronchial obstruction, and metastatic involvement of the liver and abdominal lymph nodes. Bronchoscopic biopsy followed by immunohistochemical analysis (AE1/AE3+, CD56^+^, TTF-1+, CD45^−^, LCA−) confirmed the diagnosis of primary pulmonary small cell carcinoma. Given the advanced stage of disease, systemic platinum-based chemotherapy was initiated. To provide broader clinical context, we additionally reviewed previously reported pediatric cases of PPSCC and available registry data, focusing on demographic characteristics, presenting features, diagnostic approaches, treatment strategies, and outcomes.

**Conclusion:**

This case emphasizes that PPSCC, although exceptionally uncommon, should be considered in children and adolescents with persistent respiratory symptoms, unexplained weight loss, or non-resolving pulmonary infiltrates despite appropriate treatment. Early cross-sectional imaging followed by timely tissue diagnosis is essential to avoid diagnostic delay.

## 1 Introduction

Primary lung tumors are exceptionally uncommon in children and adolescents, accounting for less than 0.2% of all pediatric malignancies [1], [2], [3]. Unlike adults, pulmonary lesions in children are far more likely to represent infectious, inflammatory, congenital, or metastatic conditions than primary epithelial lung cancers [2], [4]. Consequently, primary pulmonary malignancies are seldom considered during the initial evaluation of persistent respiratory symptoms, often resulting in delayed diagnosis.

Among pediatric primary lung cancers, primary pulmonary small cell carcinoma (PPSCC) is one of the rarest histologic subtypes [3], [5]. In adults, small cell lung cancer (SCLC) accounts for approximately 10–15% of lung cancers and is strongly associated with cigarette smoking, rapid tumor growth, early metastatic dissemination, and poor survival [5], [6]. In contrast, pediatric PPSCC has been described almost exclusively in isolated case reports and rare tumor registries, leaving its clinicopathological characteristics, optimal management, and outcomes largely undefined [7], [8], [9], [10].

The diagnosis of PPSCC is particularly challenging because its clinical manifestations are nonspecific and frequently mimic common pediatric respiratory disorders such as asthma or pneumonia [7], [11], [12]. Histopathological examination with immunohistochemistry remains the cornerstone of diagnosis, allowing differentiation from the broad spectrum of pediatric small round blue cell tumors, including lymphoma, Ewing sarcoma, rhabdomyosarcoma, and other neuroendocrine neoplasms [3], [13].

Given the extreme rarity of this tumor and the absence of pediatric-specific therapeutic guidelines, each additional well-characterized case contributes valuable information regarding diagnosis, pathological features, treatment strategies, and clinical outcomes. We report a 13-year-old boy with metastatic PPSCC presenting as persistent non-resolving respiratory disease and review the available literature to highlight the diagnostic challenges and current evidence regarding this exceptionally rare pediatric malignancy.

## 2 Case Presentation

A 13-year-old boy was referred with a 4-month history of persistent cough, mild shortness of breath, and exertional dyspnea. He was initially diagnosed with asthma and started on inhaled corticosteroids and bronchodilators; however, his symptoms showed no significant improvement. Two months later, he was admitted to the hospital with a presumed diagnosis of pneumonia and received antibiotic treatment, but his respiratory complaints continued. During this period, he had an unintentional weight loss of approximately 4 kg (12% of his body weight). More recently, he developed parasternal chest pain.

He denied fever, night sweats, or hemoptysis. Evaluation for tuberculosis, including a purified protein derivative (PPD) test and polymerase chain reaction (PCR) for *Mycobacterium tuberculosis*, was negative, and there was no history of contact with individuals with tuberculosis.

His past medical history was unremarkable, with no previous hospitalizations or chronic illnesses. He had a normal birth and developmental history and was the third child of healthy, non-consanguineous parents.

The patient's social history was notable for substantial long-term exposure to secondhand cigarette smoke, as his father was a heavy smoker who regularly smoked both inside and outside the home.

On examination, he was hemodynamically stable with an oxygen saturation of 98% while breathing room air. Respiratory examination revealed decreased breath sounds over the right mid-lung zone with localized wheezing. There was no respiratory distress, digital clubbing, or palpable peripheral lymphadenopathy.

Laboratory Findings.

Initial laboratory investigations showed elevated inflammatory markers, including an erythrocyte sedimentation rate (ESR) of 71 mm/h and positive C-reactive protein (CRP). Hyponatremia (serum sodium, 124 mEq/L) was also noted. The remaining hematologic and biochemical parameters were within normal limits or showed no clinically significant abnormalities (Table 1).

**Table 1:** Laboratory findings at presentation.

Parameter	Result	Reference Range
White Blood Cell Count (WBC)	8.7 × 10^3^/μL	4.5–11 × 10^3^/μL
Red Blood Cell Count (RBC)	4.91 × 10^6^/μL	3.6–6.1 × 10^6^/μL
Hemoglobin (Hb)	11.9 g/dL	12–16 g/dL
Hematocrit (Hct)	38.9%	36–48%
Mean Corpuscular Volume (MCV)	79.23 fL	80–96 fL
Mean Corpuscular Hemoglobin (MCH)	24.22 pg	27–31 pg
Mean Corpuscular Hemoglobin Conc. (MCHC)	30.62 g/dL	32–36 g/dL
Platelet Count (PLT)	343,000/μL	150,000–450,000/μL
Neutrophils	66.7%	40–60%
Lymphocytes	24.2%	20–40%
Monocytes	7.9%	2–8%
Eosinophils	0.1%	1–4%
C-Reactive Protein (CRP)	2+	Negative
Erythrocyte Sedimentation Rate (ESR)	71 mm/h	< 22 mm/h
Serum Ferritin	229 ng/mL	Age-appropriate range
Lactate Dehydrogenase (LDH)	443 U/L	< 1100 U/L (pediatric)
Alkaline Phosphatase (ALP)	537 U/L	50–250 U/L
Blood Urea	31 mg/dL	17–42 mg/dL
Serum Creatinine	0.6 mg/dL	0.6–1.1 mg/dL
Serum Sodium (Na^+^)	124 mEq/L	134–145 mEq/L
Serum Potassium (K^+^)	4.3 mEq/L	3.5–5.5 mEq/L
Blood Culture	Negative	No growth at 7 days
*M. tuberculosis* RT-PCR	Negative	Negative

## 3 Imaging and diagnostic workup

Chest radiographs obtained one month before and at admission showed persistent consolidation of the right middle lobe despite medical treatment (Fig. 1).

**Fig. 1:**
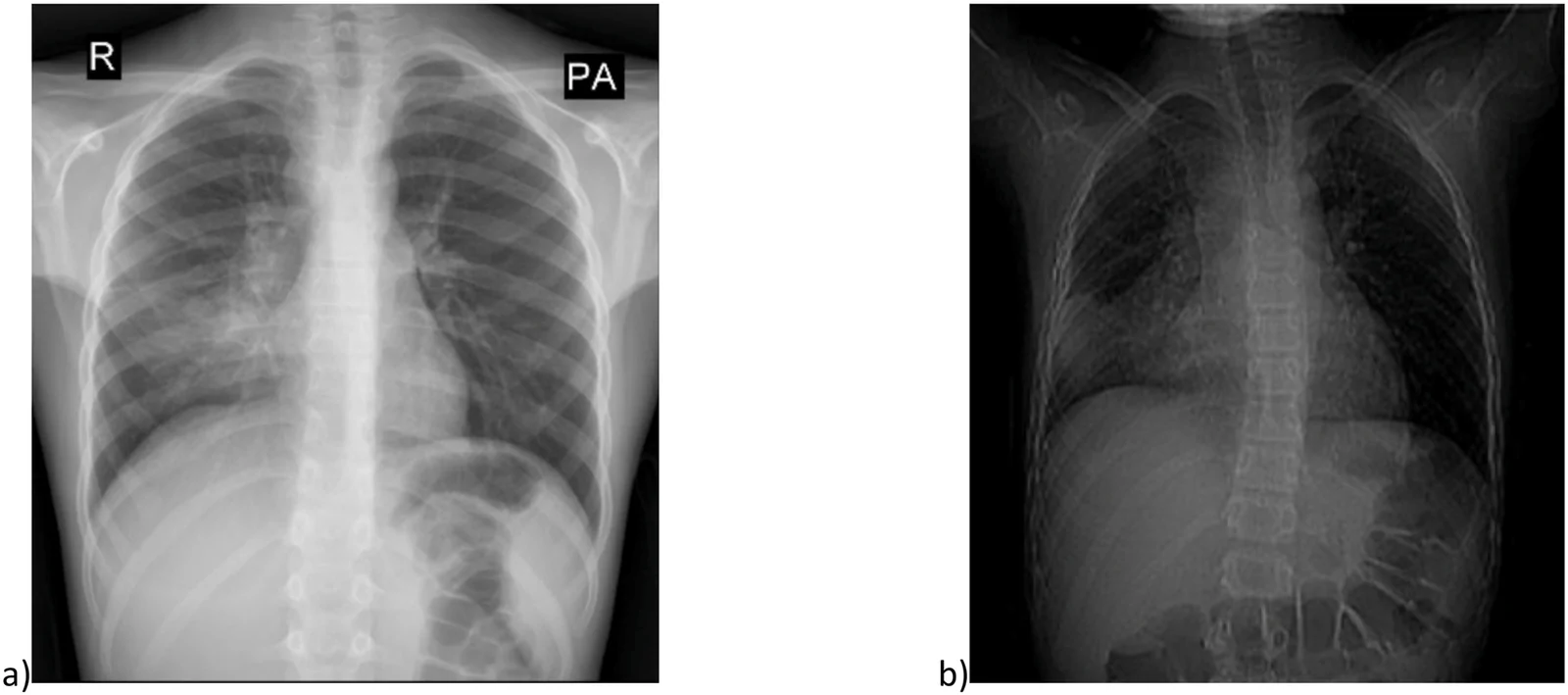
Chest X-ray Persistent right middle lobe consolidation a month before admission (a) and at admission (b).

Contrast-enhanced chest computed tomography (CT) demonstrated a large hypoenhancing conglomerate mass centered in the right hilar region with extension into both the anterior and posterior mediastinum (Fig. 2). The mass completely obstructed the right interlobar and middle lobe bronchi, causing post-obstructive pneumonia in the right upper lobe. It also markedly narrowed the lobar branches of the right pulmonary artery and partially encased the descending thoracic aorta (approximately 150°) as well as the esophagus. Several internal calcification foci were identified within the lesion. In addition, a solitary 3-mm pulmonary nodule was noted in the left upper lobe.

**Fig. 2:**
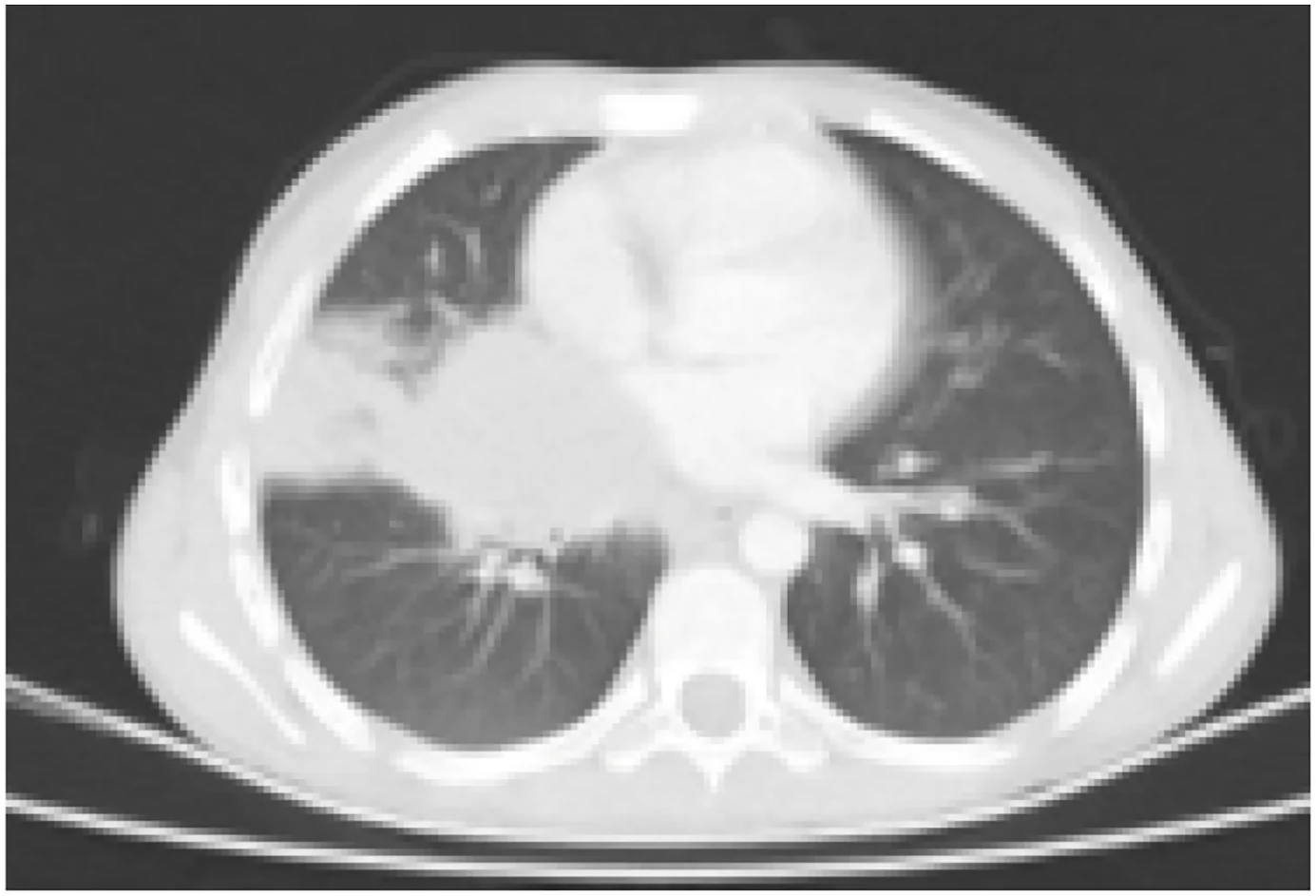
Chest CT scan demonstrating a large, conglomerated, hypo-enhancing right hilar mass extending into the mediastinum, causing obliteration of the right interlobar and middle lobe bronchi, and narrowing of the right pulmonary artery branches. Post-obstructive pneumonia involving the right upper lobe was also present.

Abdominopelvic ultrasonography revealed multiple enlarged portohepatic, peripancreatic, and para-aortic lymph nodes measuring 3–13 mm. Two hepatic lesions with a characteristic target appearance bull's-eye, the largest measuring 18 × 13 mm in segment II, were highly suspicious for metastatic disease.

Flexible bronchoscopy demonstrated a partially obstructing endobronchial lesion involving the right bronchus intermedius and extending to the right middle and lower lobe bronchi, with associated mucosal infiltration (Fig. 3). Endobronchial biopsy specimens were obtained for histopathological analysis.

**Fig. 3:**
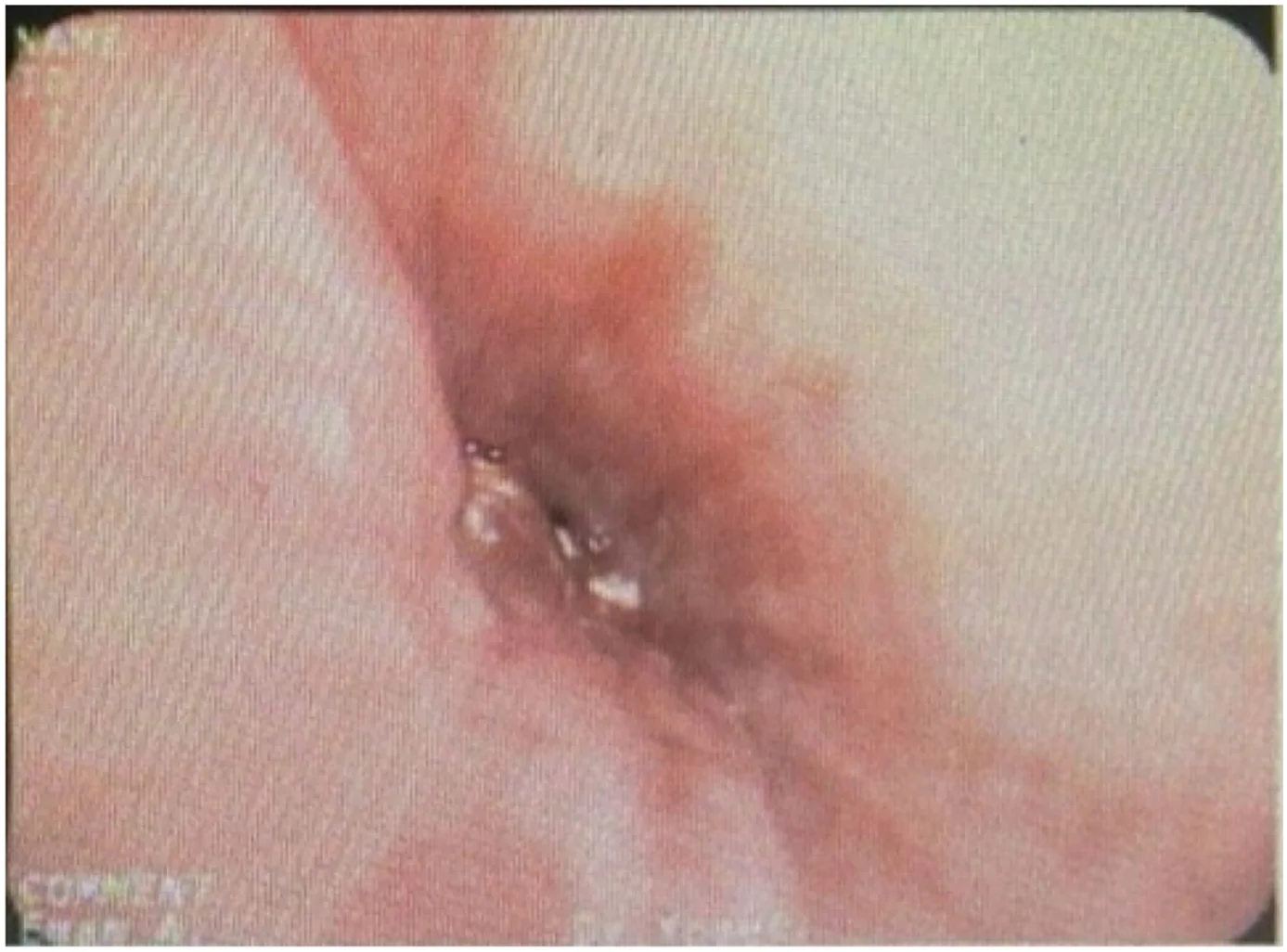
Fiberoptic Bronchoscopy Image demonstrating an endobronchial lesion within the bronchus intermedius causing partial obstruction of the right middle and lower lobe orifices, along with mucosal involvement.

## 4 Pathologic findings

Microscopic examination of the bronchoscopic biopsy demonstrated bronchial mucosa infiltrated by a malignant neoplasm composed of relatively small cells arranged in diffuse sheets. The tumor cells showed a high nuclear-to-cytoplasmic ratio, finely granular, salt-and-pepper chromatin, inconspicuous nucleoli, prominent nuclear molding, crush artifact, and focal single-cell necrosis (Fig. 4a). These findings were characteristic of small cell carcinoma.

**Fig. 4:**
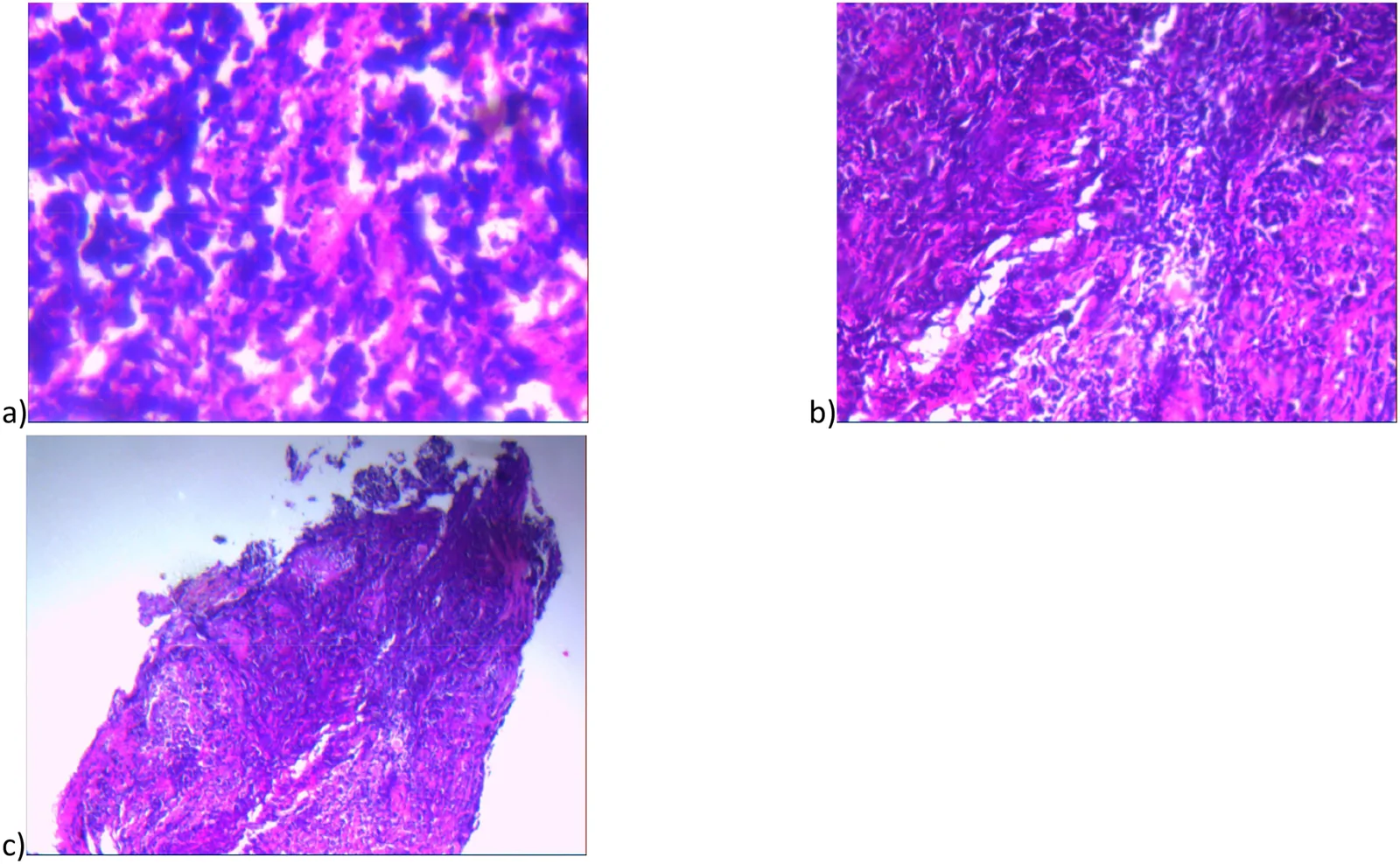
Histopathology (H&E stain) Microscopic examination demonstrating sheets of small malignant cells with hyperchromatic nuclei, scant cytoplasm, and nuclear molding consistent with small cell carcinoma. Magnification: (a) ×40, (b) ×10, and (c) ×4.

Immunohistochemical analysis showed punctate positivity for cytokeratin AE1/AE3, diffuse membranous expression of CD56, and weak nuclear staining for thyroid transcription factor-1 (TTF-1). Napsin A was negative. Hematolymphoid markers, including CD45, CD20, CD3, and terminal deoxynucleotidyl transferase (TdT), were also negative, excluding lymphoma and lymphoblastic neoplasms (Fig. 5).

**Fig. 5:**
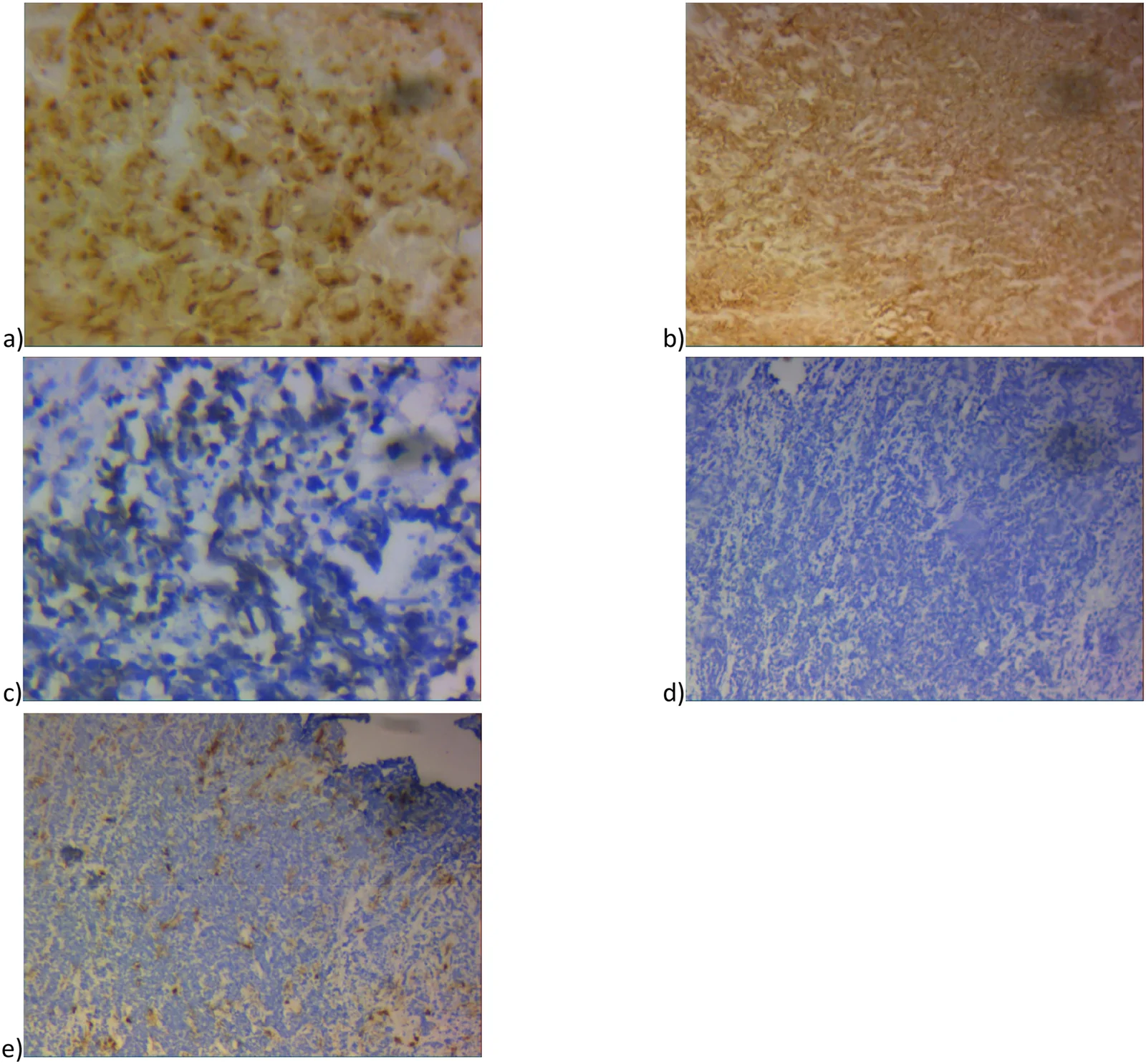
Immunohistochemical findings in pulmonary small cell carcinoma. (a) Chromogranin A, (b) synaptophysin, (c) thyroid transcription factor-1 (TTF-1), (d) CD45, and (e) pancytokeratin (AE1/AE3)

Taken together, the morphologic and immunophenotypic findings established the diagnosis of primary pulmonary small cell carcinoma.

## 5 Treatment and outcome

Following diagnosis, the patient was referred to the pediatric oncology service. Given the extensive mediastinal involvement and radiologic findings highly suggestive of hepatic metastases, the disease was managed as advanced-stage small cell lung carcinoma. Chemotherapy was initiated according to the institutional pediatric oncology protocol consisting of vincristine, doxorubicin, cyclophosphamide, methylprednisolone, and MESNA. The first and second treatment cycle was well tolerated without major complications, and the patient was discharged with plans for continuation of chemotherapy and close outpatient follow-up.

## 6 Discussion

Primary pulmonary small cell carcinoma (PPSCC) is an exceptionally rare pediatric thoracic malignancy. Large pediatric registries and institutional series spanning several decades have reported either no cases or only a handful of patients with PPSCC (Table 2). [8], [9], [10], [14], [15], [16], [17] Consequently, current knowledge is derived almost entirely from isolated case reports. (Table 3) [8], [9], [10], [14], [15], [16], [17].

**Table 2:** Major pediatric lung carcinoma registries and case series reporting primary pulmonary small cell carcinoma.

Study/Registry	Study Period	Total Primary Pediatric Lung Cases	Total Malignant Cases	SCLC/PPSCC Cases	Main Findings & Pathology Distribution
**Dishop & Kuruvilla (2008)**[15]	1982 – 2007	34 cases	14 cases	**0 cases**	PPB and Carcinoid were the most common malignancies. No SCLC cases were reported.
**Yu et al. (2010)**[18]	1918 – 2008	40 cases	25 cases	**1 case**	Carcinoid and IMT were the most common histologies. Only 1 SCLC case was reported in an adolescent male.
**Rojas et al. (2015)**[19]	1998 – 2011	211 cases (Epithelial)	211 cases	**2 cases**	Carcinoid was the most common histology. Both SCLC patients deceased within 5 years of follow-up.
**Giuseppucci et al. (2016)**[16]	1990 – 2014	38 cases	25 cases	**0 cases**	IMT, Carcinoid, and PPB were the most frequent histologies. No SCLC cases were identified.
**Youlden et al. (2020)**[20]	1983 – 2015	53 cases	53 cases	**0 cases**	Pleuropulmonary blastoma (PPB) and Carcinoid were the most common. No SCLC cases reported.
**Voggel et al. (2021)**[9]	2009 – 2019	12 cases	12 cases	**0 cases**	Mucoepidermoid carcinoma (MEC) was the most frequent entity. No SCLC cases were identified.
**Abele et al. (2022)**[8]	2000 – 2021	38 cases	38 cases	**1 case**	Mucoepidermoid carcinoma (MEC) was the most common. Only 1 SCLC case was reported.

**Table 3:** Reported cases of primary pulmonary small cell carcinoma in children and adolescents: Clinical characteristics, treatment, and outcomes.

Reference	Age(Years)/Sex	Clinical Presentation	Smoking History	Disease Extent	Treatment	Outcome
**Kim et al. (2000)**[7]	14/M	Cough, hemoptysis, fever; pulmonary mass with pneumonitis	Not reported	Limited-stage disease (localized pulmonary lesion)	Chemotherapy + radiotherapy	Partial response (∼50% tumor reduction); long-term outcome not reported
**Robinet et al. (1994)**[14]	17/M	Respiratory symptoms; bronchial small cell carcinoma	Not reported	Not clearly reported	Not clearly reported	Outcome not available
**Tronnes et al. (2012)**[11]	14/F	Chronic cough, fatigue, decreased appetite, weight loss, chest pain	No smoking history	Limited-stage SCLC with subcarinal lymph node involvement	Chemotherapy (cisplatin/etoposide), surgery, postoperative chemoradiotherapy	Local and brain relapse; died 21 months after diagnosis
**Yonemori et al. (2006)**[12]	18/F	Asymptomatic; mass detected on routine chest radiography	Never smoker	Limited-stage SCLC (T1N2M0, stage IIIA)	Concurrent cisplatin/etoposide + thoracic radiotherapy; salvage pneumonectomy	Alive without recurrence 13 months after salvage surgery
**Barbour et al. (2018)**[21]	15/M	Chest pain, weight loss, dyspnea	Significant passive smoke	Extensive-stage disease (T3N2M1b) with liver and bone metastases	Cisplatin/etoposide; second-line gemcitabine	Died 19 months after diagnosis
**Post et al. (2012)**[13]	16/F	Cough, fever, night sweats, extremity pain	Not reported	Disseminated neuroendocrine carcinoma with liver, lung, kidney, bone marrow	Multi-agent chemotherapy (including ICE); trabetedin trial	Progressive disease; died after trial withdrawal
**Yu et al. (2010)**[18]	15/M	Asthma, cough, dyspnea	Never smoker	Localized hilar and mediastinal involvement	Systemic Chemotherapy & Radiotherapy	Alive on therapy
**Abele et al. (2022)**[8]	15.8/M	Cough, dyspnea, subcutaneous neck nodule	Unspecified	Stage IVA (Subcutaneous neck metastasis)	Lobectomy, RT (70 Gy), 1st-line Chemo (Cisplatin + Etoposide), subsequent lines (Lurbinectedin, Carboplatin/Atezolizumab)	Disease progression at 1.5-year follow-up
**Current case**	13/M	Persistent cough, dyspnea, weight loss; treated as asthma/pneumonia	Heavy chronic passive paternal cigarette smoke	Advanced disease with mediastinal invasion, abdominal lymphadenopathy, hepatic metastases	Pediatric oncology chemotherapy protocol (vincristine, doxorubicin, cyclophosphamide, methylprednisolone, MESNA)	Under treatment

The present case highlights the diagnostic challenges associated with PPSCC in children. Our patient initially presented with persistent cough, exertional dyspnea, and weight loss and was treated for asthma and subsequently pneumonia before the underlying malignancy was identified. Similar diagnostic delays have been described in most published pediatric cases because the initial manifestations closely resemble common respiratory illnesses [7], [11], [20], [21]. This experience emphasizes that persistent respiratory symptoms, particularly when accompanied by constitutional features or unresolved radiographic abnormalities, should prompt reconsideration of the diagnosis. Early chest CT and timely bronchoscopic biopsy may help establish the diagnosis before extensive disease develops.

The radiological and pathological findings in our patient were consistent with the aggressive nature of SCLC that closely parallels previous reports showing that PPSCC commonly presents as a centrally located tumor with early regional and distant spread [3], [6], [7], [8], [19], [20], [21]. Histopathological examination, supported by the characteristic immunophenotype of AE1/AE3, CD56, and TTF-1 positivity together with negative hematolymphoid markers, established the diagnosis in accordance with current WHO criteria [3].

One of the most interesting findings in our patient was severe hyponatremia at presentation (124 mEq/L). In adult SCLC, syndrome of inappropriate antidiuretic hormone secretion (SIADH) is the most frequent paraneoplastic endocrine syndrome, occurring in approximately 10–15% of patients and typically presenting with hyponatremia [22]. Although biochemical confirmation of SIADH was not available, no alternative explanation for the electrolyte disturbance was identified, raising the possibility of occult paraneoplastic SIADH [3], [22]. To our knowledge, this feature has rarely been emphasized in pediatric PPSCC and may represent an additional clue in children presenting with persistent pulmonary disease accompanied by otherwise unexplained hyponatremia.

The etiology of pediatric PPSCC remains uncertain and is likely distinct from that of adult SCLC, which is strongly associated with active cigarette smoking [5], [6]. Our patient had prolonged exposure to second-hand tobacco smoke because of heavy paternal smoking within the household. Although a causal relationship cannot be inferred from a single case, epidemiological studies have consistently linked passive smoking to chronic airway injury and an increased risk of lung cancer [23], [24]. Similar exposure has also been documented in at least one previously reported pediatric PPSCC case [20]. Whether environmental tobacco smoke contributes to pulmonary neuroendocrine carcinogenesis during childhood warrants further investigation.

Given the rarity of PPSCC, treatment in children continues to be extrapolated largely from adult SCLC protocols [6], [8], [24]. Our patient presented with presumed metastatic disease and was managed with systemic chemotherapy according to our institutional pediatric oncology protocol. Nevertheless, outcomes remain poor, largely because most pediatric patients are diagnosed at an advanced stage [8], [19], [20], [21]. Increasing awareness of this rare entity among pediatricians, pulmonologists, and radiologists may facilitate earlier diagnosis and improve the opportunity for timely treatment.

This case contributes to the limited literature by documenting a child with advanced PPSCC, suspected hepatic metastases, marked hyponatremia suggestive of possible paraneoplastic SIADH, prolonged second-hand tobacco smoke exposure, and characteristic clinicopathological findings confirming the diagnosis. Although conclusions regarding causality or optimal treatment cannot be drawn from a single observation, accumulating well-documented cases through international collaborative registries will be critical for defining the biological characteristics, prognostic factors, and optimal management of this exceptionally rare pediatric malignancy.

## CRediT authorship contribution statement

**Seyedeh Zalfa Modarresi:** Conceptualization, Data curation, Investigation, Methodology, Project administration, Resources, Supervision, Visualization, Writing – original draft, Writing – review & editing. **Mahla Towhidifar:** Conceptualization, Data curation, Investigation, Writing – original draft, Writing – review & editing. **Somayeh Talaeipour:** Conceptualization, Data curation, Validation, Writing – original draft, Writing – review & editing. **Maryamsadat Yazdanparast:** Data curation, Supervision, Writing – review & editing. **Fariba Binesh:** Conceptualization, Data curation, Supervision, Writing – review & editing. **FatemehalSadat AghaeiMeybodi:** Conceptualization, Data curation, Investigation, Methodology, Writing – review & editing.

## Ethical approval

This case report was conducted in accordance with the ethical standards of the institutional and national research committee and with the principles of the Declaration of Helsinki. Approval from the Research Ethics Committees of Shahid Sadoughi University of Medical Sciences **(IR.SSU.REC.1405.126)**

## Consent for publication

Written informed consent for publication of the clinical details and accompanying images was obtained from the patient's parents guardians.

## Data availability statement

All data generated or analyzed during this study are included in this published article. Additional information may be obtained from the corresponding author upon reasonable request.

## Funding

No fund was available for this case report.

## Declaration of competing interest

The authors declare that they have no known competing financial interests or personal relationships that could have appeared to influence the work reported in this paper.
